# Impact of administration route on nanocarrier biodistribution in a murine colitis model

**DOI:** 10.1080/17458080.2022.2134563

**Published:** 2022-10-19

**Authors:** Catherine C. Applegate, Hongping Deng, Brittany L. Kleszynski, Tzu-Wen L. Cross, Christian J. Konopka, L. Wawrzyniec Dobrucki, Erik R. Nelson, Matthew A. Wallig, Andrew M. Smith, Kelly S. Swanson

**Affiliations:** a Division of Nutritional Sciences, University of Illinois at Urbana – Champaign, Urbana, Illinois, USA;; b Department of Animal Sciences, University of Illinois at Urbana – Champaign, Urbana, Illinois, USA;; c Department of Bioengineering, University of Illinois at Urbana – Champaign, Urbana, Illinois, USA;; d Department of Pathobiology, College of Veterinary Medicine, University of Illinois at Urbana – Champaign, Urbana, Illinois, USA;; e Department of Nutrition Science, Purdue University, West Lafayette, Indiana, USA;; f Beckman Institute for Advanced Science and Technology, Urbana, Illinois, USA;; g Cancer Center at Illinois, University of Illinois at Urbana – Champaign, Urbana, Illinois, USA;; h Department of Molecular and Integrative Physiology, University of Illinois at Urbana – Champaign, Urbana, Illinois, USA;; i University of Illinois Cancer Center, University of Illinois at Chicago, Chicago, Illinois, USA;; j Carl R. Woese Institute for Genomic Biology, Anticancer Discovery from Pets to People Theme, University of Illinois at Urbana – Champaign, Urbana, Illinois, USA;; k Carle Illinois College of Medicine, Urbana, Illinois, USA;; l Micro and Nanotechnology Laboratory, University of Illinois at Urbana – Champaign, Urbana, Illinois, USA;; m Department of Materials Science and Engineering, University of Illinois at Urbana – Champaign, Urbana, Illinois, USA

**Keywords:** inflammatory bowel disease, colitis, PET, nanoparticles, dextran

## Abstract

The incidence of inflammatory bowel disease (IBD) is increasing worldwide. Although current diagnostic and disease monitoring tests for IBD sensitively detect gut inflammation, they lack the molecular and cellular specificity of positron emission tomography (PET). In this proof-of-concept study, we use a radiolabeled macrophage-targeted nanocarrier probe (^64^Cu-NOTA-D500) administered by oral, enema, and intraperitoneal routes to evaluate the delivery route dependence of biodistribution across healthy and diseased tissues in a murine model of dextran sodium sulfate (DSS)-induced colitis. High inter-subject variability of probe uptake in intestinal tissue was reduced by normalization to uptake in liver or total intestines. Differences in normalized uptake between healthy and DSS colitis animal intestines were highest for oral and IP routes. Differences in absolute liver uptake reflected a possible secondary diagnostic metric of IBD pathology. These results should inform the preclinical development of inflammation-targeted contrast agents for IBD and related gut disorders to improve diagnostic accuracy.

The incidence of inflammatory bowel disease (IBD), including Crohn’s disease and ulcerative colitis, is increasing worldwide, with the United States showing the highest prevalence rate [1]. IBD is characterized by chronic, idiopathic, and relapsing inflammation of the mucosal lining of the gastrointestinal (GI) tract. If left untreated, this chronic inflammation leads to GI distress and tissue damage, lowering patient quality of life and potentially resulting in more serious complications such as strictures, abscesses, and fistulas as well as an increased risk for colon cancer [2]. While the etiology of IBD remains unknown, a wealth of accumulating evidence suggests that aberrant infiltration and activation of proinflammatory immune cells is the driving force of disease pathogenesis [3, 4].

The diagnosis of IBD typically requires invasive colonoscopy and biopsy for histopathological confirmation of intestinal inflammation, with magnetic resonance imaging (MRI) and X-ray fluoroscopy or computed tomography (CT) used to evaluate the extent of the disease and to monitor disease activity [5]. Although highly sensitive and high in resolution, MRI and CT lack the specificity of positron emission tomography (PET), which can be used to quantitatively assess inflammation throughout the whole gut, anatomically localized by correlation with CT imaging [6]. Radiolabeled fluorodeoxyglucose (^18^F-FDG) can be used to identify inflammatory lesions by PET/CT imaging; however, non-specific uptake of FDG in the healthy bowel can lead to false positive results, and the probe is more a direct measure of metabolism than inflammation [7]. PET detection of radiolabeled autologous leukocytes that home to the site of intestinal inflammation offers improved specificity compared with FDG, but this method requires specialized handling of patient blood for leukocyte isolation, radiolabeling, and reinjection [8].

Nanoparticles have been widely investigated as contrast agents for imaging-based evaluation of disease extent [9–11] and for delivering colon-targeted therapies for IBD [12–16]. However, few studies have examined the potential to detect intestinal inflammation using radiolabeled nanoparticles for high-specificity PET imaging [17]. Furthermore, it is not clear which administration route would be best suited for delivery of inflammation-targeting nanomaterials in IBD to maximize the contrast between inflamed and healthy intestines. Nanoparticles natively distribute to healthy tissues [18], and uptake specificity in inflamed tissues depends on the route of administration [19]. In the intestines, the luminal surface is inflamed in IBD and is directly accessible *via* oral and enema administrations, while the visceral membrane is accessible through intraperitoneal (IP) administration. Systemic administration (e.g. intravenous) can transport nanomaterials to intestinal tissues; however, uptake in inflamed tissue derives from vascular permeability rather than specific interstitial cell populations, and the vast majority of material distributes to organs of the mononuclear phagocytotic system (liver and spleen) [17, 20].

The goal of this study was to evaluate the impact of administration route on the distribution of nanocarriers to inflamed intestinal tissue in a rodent model of colitis. We focused on a polysaccharide (dextran) nanocarrier due to its specific uptake by macrophages, cells that are prevalent in inflamed intestines. We previously used the same material conjugated to a copper (Cu) radiolabel to target inflamed visceral adipose depots of obese mice and found that >90% of the 500 kDa nanocarrier was taken up by adipose tissue (AT) macrophages [21]. In addition to their macrophage targeting ability, polysaccharides such as dextran are advantageous to use as nanocarriers due to their low cost, biocompatibility, ease of chemical conjugation, and long record of clinical safety for use both as a blood volume expander and to reduce platelet aggregation [22]. In this study, we investigated the differences between healthy and inflamed tissue biodistributions of a dextran-based nanocarrier conjugate across oral, enema, and IP routes of administration. Through PET/CT imaging and quantitative radioisotopic analysis, we found that IP delivery resulted in the highest nanocarrier retention across all measured tissues of both healthy animals and animals with dextran sodium sulfate (DSS)-induced colitis. Few intestinal tissues were statistically distinguishable between the healthy and DSS colitis animal groups for any of the administration routes due to a high degree of inter-subject variability in absolute distribution to intestinal tissues. This variability was reduced by ratiometric normalization to liver or total intestinal uptake to reveal significant group differences, especially for oral and IP administration routes. IP delivery resulted in higher levels of nanocarrier accumulation within liver tissues of DSS colitis animals, which may indicate disease-specific hepatic processes that can be probed independently by these contrast agents. These findings can inform the design of larger scale studies aimed to evaluate longitudinal progression or therapeutic response in IBD using PET/CT.

## Materials and methods

### Synthesis of ^64^Cu-NOTA-D500

Dextran conjugates of the chelator 1,4,7-triazacyclononane-1,4,7-triacetic acid were synthesized from aminated 500 kDa dextran (D500-NH_2_) as reported previously [21]. D500-NH_2_ (10 mg) was dissolved in 5 mL anhydrous dimethylsulfoxide (DMSO) and anhydrous triethylamine (10 μL) was added. A solution of *p*-SCN-Bn-NOTA (0.68 mg) in 1 mL anhydrous DMSO was added, and the mixture was stirred at room temperature for 16 hr. The product was purified using an Amicon filter (MWCO 30 kDa), and the solid product was collected after lyophilization. To label with 64Cu, a solution of NOTA-D500 in sodium acetate buffer (pH = 5.5, 0.1 M) was mixed with a solution of ^64^CuCl_2_ (Washington University, St. Louis, MO, USA). The mixture was incubated for 30 min at 37 °C before nonradioactive CuCl_2_ (equivalent molar amount to NOTA in the dextran conjugate) was added to saturate unreacted NOTA. The mixture was incubated for 20 min and ethylenediaminetetraacetic acid (EDTA, 2 equivalents to total Cu) was added to scavenge nonspecifically bound and/or free Cu. After incubation for 10 min, free Cu, EDTA, and EDTA-Cu chelates were removed using an Amicon filter (MWCO 30 kDa), and buffer was exchanged to phosphate buffered saline (PBS). Radiochemical purity (RCP) was determined by thin-layer chromatography, and samples with RCP > 95% were used for imaging experiments.

### Animals and diets

All animal procedures were approved by the University of Illinois Institutional Animal Care and Use Committee prior to experimentation (protocol #17087). Six-week old male C57Bl/6J mice (Jackson Laboratory, Bar Harbor, ME, USA) were purchased and housed individually in standard shoebox cages in a temperature- and humidity-controlled room, with a 12 hr light/dark cycle. Upon arrival, mice were fed a low-fat diet (10% kcal from fat; Research Diets Inc. #D12450J, New Brunswick, NJ, USA) *ad libitum* with free access to fresh water and allowed to acclimate to the facility for 2 wk prior to DSS treatment regimen.

### DSS treatment

Previous studies showed that providing 3% DSS water for 5 d is sufficient to induce colitis in C57Bl/6J mice, with similar pathology to UC in humans [23, 24]. After acclimation, mice were randomly assigned to receive distilled water (control; Con) or water containing DSS (DSS) for 5 d (d 1–5) to induce acute colitis. For animals treated with DSS, regular drinking water was replaced with 3% (wt:vol) DSS (36,000–50,000 kDa molecular weight; MP Biochemicals, Santa Ana, CA, USA) in sterile water. Freshly made DSS-water was provided on d 1, 3, and 5, and fecal scores were assessed daily to monitor colitis. Fecal scores were assigned based on stool consistency and the presence of occult blood, as previously described [25], and recorded daily using fresh (within 15 min of defecation) fecal samples. A hemoccult kit (Beckman Coulter, Brea, CA, USA) was used to determine the presence of occult blood in stool. Following DSS treatment, all mice were provided with fresh, untreated water for the remaining duration of the study.

### Biodistribution studies

To determine the optimal nanocarrier administration route, mice were administered ^64^Cu-NOTA-D500 (~100 μCi) by oral, IP, or enema routes, 2 days after completing DSS treatment (d 8). Con and DSS mice were both used (*n* = 3/group). For oral administration, 100 μL of nanocarrier in PBS was administered by oral gavage to unanesthetized mice. For IP administration, unanesthetized mice were injected with 100 μL of nanocarrier in PBS on the left side. For enema administration, mice were anesthetized by isoflurane and infused with 100 μL of nanocarrier in PBS. Mice were euthanized by CO_2_ asphyxiation and cervical dislocation 24 hr following nanocarrier treatment. Blood was collected by cardiac puncture, and tissues were collected including adipose depots (subcutaneous, gonadal, perirenal, mesenteric), liver, spleen, stomach, and intestines (sectioned by duodenum, jejunum, ileum, proximal colon, mid colon, distal colon, and cecum). Radioactivity in tissues was measured using a Wizard2 Automatic γ-counter (PerkinElmer, Waltham, MA, USA) as previously described [21]. One mouse from each group was randomly selected to undergo serial live PET/CT imaging at 4 and 24 hr following conjugate delivery using an Inveon PET/CT system (Siemens Healthcare, Malvern, PA, USA) as previously described [21]. CT contrast of the intestines was provided by enema-delivered iohexol (GE Healthcare, Chicago, IL, USA).

### Histopathology

Mid-colon tissues were fixed in 10% neutral-buffered formalin for 24 hr and then transferred to 70% ethanol until embedding in paraffin (Tissue Tek VIP, Sakura Finetek USA, Inc., Torrance, CA, USA). Tissues were sectioned into 7 μm-thick slices using a micro-tome (Microm HM 310, MICROM Laborgeräte GmbH, Berlin, Germany) and mounted onto glass slides (Surgipath^®^ R X-Tra^®^ R Microscope Slides, Leica Biosystems, Buffalo Grove, IL, USA). Mounted sections were stained with Harris’ hematoxylin and eosin (H&E), and sections were blindly evaluated for the presence of colitis by a board-certified veterinary pathologist at the University of Illinois (MAW).

### Statistical analysis

Biodistribution data were analyzed between treatment groups within each mode of delivery (Wilcoxon Rank Sum) and between modes of delivery (Kruskal-Wallis with post hoc analysis by the Dwass, Steel, Critchlow-Fligner method) using SAS (v.9.4, SAS Institute, Cary, NC, USA). Statistical significance was set as *p* < 0.05 and a trend was set as *p* < 0.10.

## Results

### Dextran nanocarrier radiochelate: ^64^Cu-NOTA-D500

**Figure 1.**
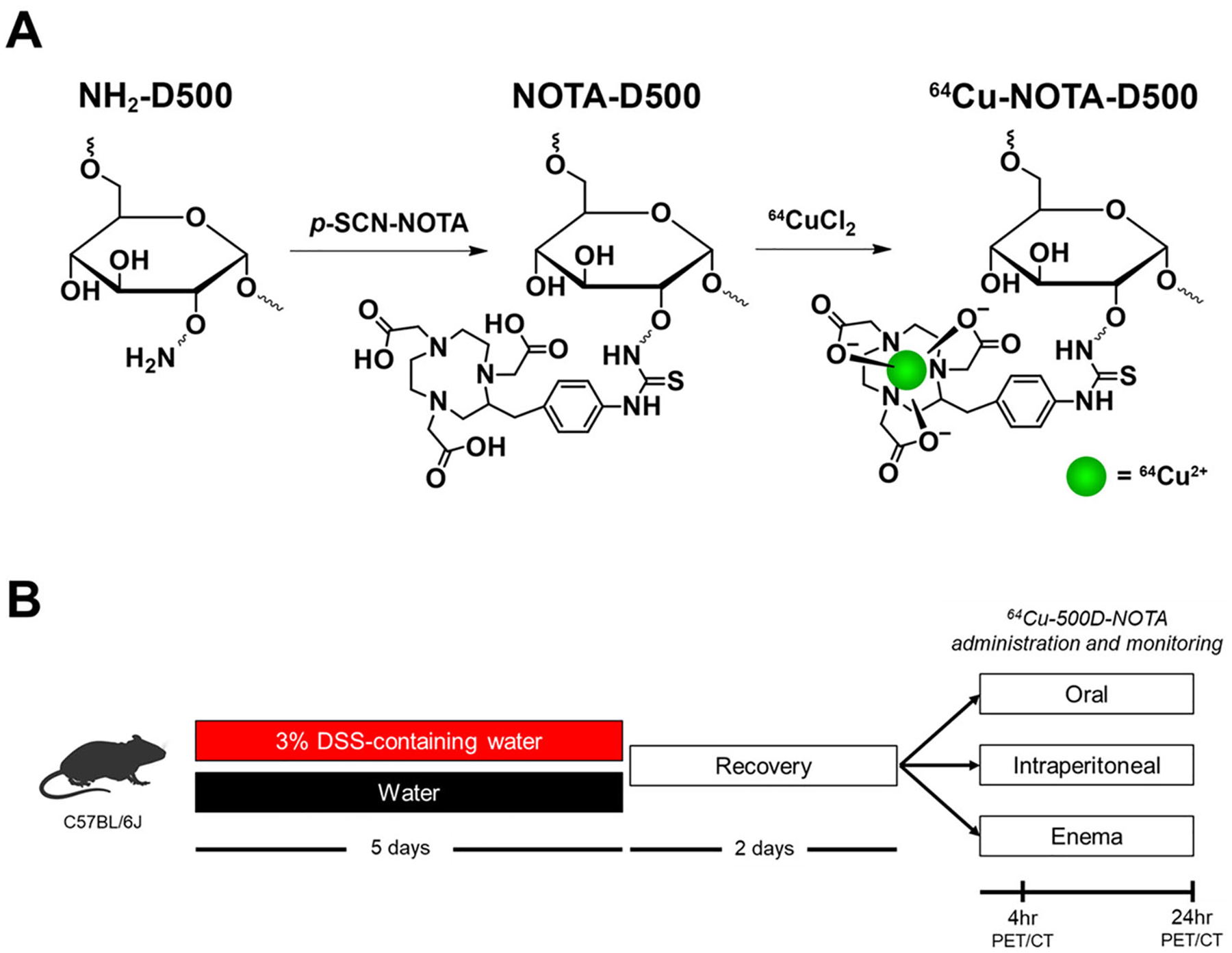
Dextran conjugates and experimental workflow. (A) Chemical structures of 500 kDa molecular weight dextran (D500), NOTA-D500 conjugate, and ^64^Cu-NOTA-D500 probe. (B) Study design for treatment of DSS colitis animals and healthy controls.

The synthesis scheme for ^64^Cu-NOTA-D500 is shown in Figure 1A. Polysaccharides such as dextran are selectively internalized by macrophages through binding with plasma membrane C-type lectins and class A scavenger receptors [26, 27]. Our previous work demonstrated efficient uptake of dextran polysaccharides of high molecular weight (500 kDa) by macrophages both *in vitro* and *in vivo*, with higher specificity for uptake by M1 macrophages present within inflamed AT in a murine model of obesity [21]. We therefore synthesized contrast agents using 500 kDa dextran derived from aminated commercial biopolymers, linked to NOTA through stable thiourea bonds. The dextran amines were saturated by excess *p*-SCN-NOTA (2.5:1 to amines) with base catalysis and purified. Conjugation was confirmed *via* proton nuclear magnetic resonance (^1^H NMR) spectroscopy (Figure S1), showing the appearance of peaks at 7.0–7.3 ppm that were consistent with NOTA. The number of NOTA groups per dextran was near 92 based on ICP-MS elemental analysis of Cu after reaction with excess CuCl_2_ and purification by filtration. To prepare the radiochelate, ^64^CuCl_2_ was added to NOTA-D500 in acetate buffer (pH 5.0) before saturation of NOTA with excess cold Cu, followed by repeated purification by filtration.

### Dependence of absolute ^64^Cu-NOTA-D500 biodistribution on administration route

**Figure 2.**
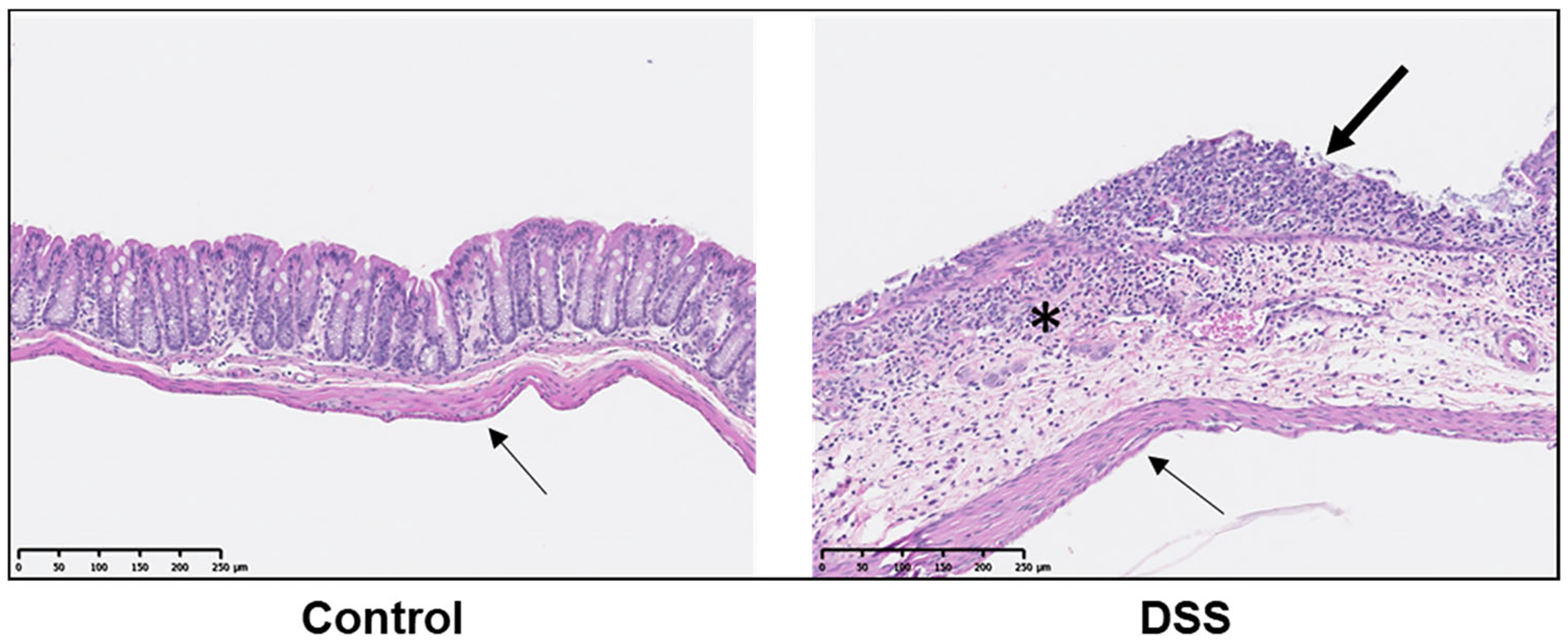
Histopathological micrographs of normal and inflamed colons. Control animals display an intact lining of the colon. In animals with DSS-induced colitis, erosive lesions are present with complete loss of mucosal epithelium (thick arrow), collapse of mucosa, and inflammatory cell infiltrate into the submucosa (asterisk). In both animal tissues, there is a single layer of elongated mesothelial cells closely apposed to the outer tunica muscularis (thin arrows), demonstrating an intact mesothelium.

Mice were treated with 3% DSS in water for 5 d to induce a colitis-like phenotype [23, 24] or treated with plain water (Con). Animals were allowed to recover for 2 d and were randomized within treatment groups to receive a single dose of radiolabeled probe (^64^Cu-NOTA-D500) by either oral gavage, enema, or IP injection (*n* = 3/group; Figure 1B). Colitis was monitored by presence of occult fecal blood, and extensive colitis was observed in both the colon and cecum of all mice, as evaluated by a board-certified veterinary pathologist (MAW) *post mortem*. Colitis in both animal models and humans is associated with a disruption in the mucus layer of the GI tract at the site of inflammation, leading to mucosal collapse [28–30], as verified in our histologic observations (Figure 2). A healthy mucus barrier may trap nanoparticles [31], as polymeric particles can bind to proteins present within the mucus [32]. Alternatively, loss of mucosa in colitis may be associated with more rapid probe diffusion, uptake, and/or clearance.

**Figure 3.**
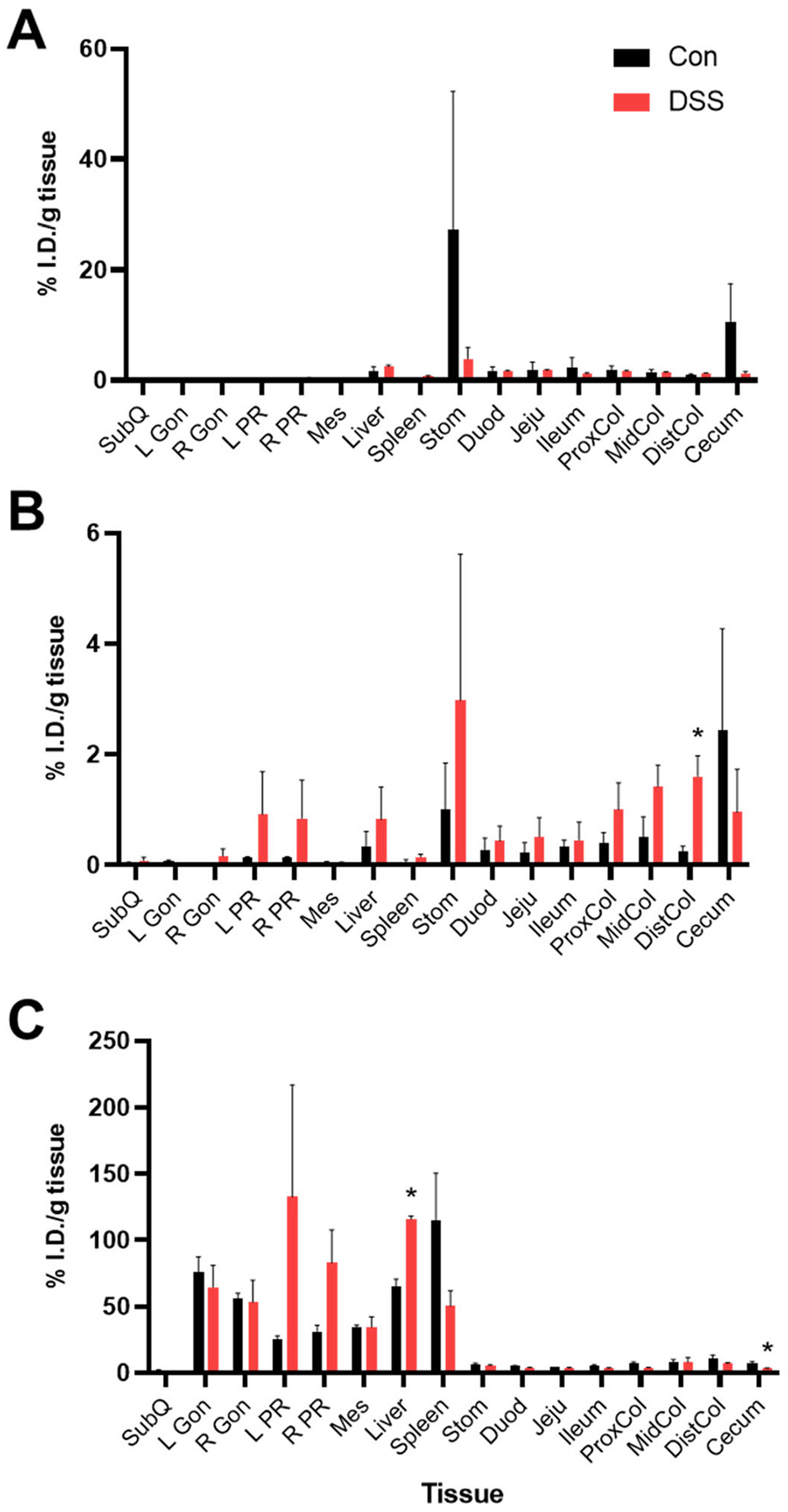
Biodistribution of ^64^Cu-NOTA-D500 differs by mode of administration in control (Con) and animals with colitis (DSS). Quantitative gamma well counting results 24 hr following (A) oral; (B) enema; or (C) intraperitoneal (IP) administration of ^64^Cu-NOTA-D500. Data are shown as mean ± SEM of the % injected dose per gram of tissue (I.D./g), *n* = 3/group. *Indicates significantly different from Con (*p* < 0.05 by Wilcoxon Rank Sum). Abbreviations: subcutaneous (SubQ), left/right gonadal (L/R Gon), left/right perirenal (L/R PR), and mesenteric (Mes) adipose depots; stomach (Stom); duodenum (Duod); jejunum (Jeju); proximal colon (ProxCol); mid colon (MidCol); distal colon (DistCol).

Twenty-four hours after ^64^Cu-NOTA-D500 administration, *post mortem* gamma well counting (GWC) was used to evaluate tissue uptake of the probe in excised tissues, including intestines, stomach, liver, spleen, and AT depots. Animals administered with oral gavage showed detectable probe levels in all tissues of the GI tract as well as the liver (Figure 3A). Complete data are provided in Table 1. Retention in the stomach and total intestines was higher on average for Con mice compared with DSS mice, although absolute values were highly variable, so these differences were not statistically significant (27.2% vs. 3.9% and 20.5% vs. 10.3% of the injected dose per gram [I.D./g] tissue, respectively). This effect may be due to decreased gut transit time with IBD or degradation by microbiota-derived dextranases during 64Cu-NOTA-D500 passage through the digestive system [33]. Overall, retention in individual tissues of the intestine, where inflammation is observed with colitis, was low (<2%) and not statistically different between Con and DSS mice.

Enema administration led to significantly less uptake by the small intestines (duodenum, jejunum, ileum) compared with oral administration (Figure 3B). Higher mean retention was measured throughout intestinal tissues of DSS mice (6.4% I.D./g) compared with those of Con mice (4.4% I.D./g), which was opposite to that of oral gavage. Intestinal uptake differences between the DSS and Con mice were only statistically significant in the distal colon (1.6% for DSS mice vs. 0.2% I.D./g for Con mice), which is the primary site of DSS-induced colitis [23].

IP administration of ^64^Cu-NOTA-D500 led to significantly greater absolute retention throughout the body 24 hr post-injection in comparison to oral and enema delivery modes (Figure 3C). As apparent from the PET/CT images, a large proportion of the I.D. localized to the liver and AT in both Con and DSS animals. Interestingly, IP injection resulted in significantly higher probe uptake in livers of DSS-treated animals (116.0% I.D./g) compared with livers of Con animals (65.0% I.D./g), while uptake in the spleen followed the opposite pattern. The liver and spleen contain the highest concentrations of macrophages and are thus the sites of dextran biodistribution after systemic administration [34]. The liver is strongly affected during IBD progression, with up to 50% of patients also experiencing some form of hepatobiliary disease [35]. As such, increased liver uptake of the probe in DSS mice may reflect an increased population of liver macrophages in the inflamed colitis state, redirecting ^64^Cu-NOTA-D500 from the spleen to the liver.

Absolute retention by the intestines was significantly higher following IP administration compared with oral gavage or enema. Overall intestinal distribution trended higher for Con animals (48.7% I.D./g) compared with DSS-treated animals (32.6% I.D./g). These differences were statistically significant within the cecum (7.6% vs. 3.1% I.D./g) but were only trending within the proximal colon (7.1% vs. 3.8% I.D./g). It was not determined if intestinal uptake was due to direct uptake across the visceral mesothelium of the intestines or as a result of hepatobiliary excretion [36], although an intact mesothelium in both inflamed and non-inflamed colons was evident from histopathology (Figure 2), so the latter is more likely. Therefore, while both IP injection and oral gavage led to superior absolute probe uptake and retention in intestines relative to enema administration, only enema delivery led to greater disease-specific probe retention at the site of colitis-associated inflammation (colon) in DSS animals compared with healthy Con animals.

### Relative tissue uptake: liver normalization

High variability of biodistribution is an expected outcome for the three administration routes explored here for which the probe solution is initially dispersed to the surface or lumen of the GI tract, which is itself variable, heterogenous in contents, and undergoing peristalsis [37]. A useful approach to addressing this type of variability is to calibrate the target tissue uptake based on probe dose in a secondary tissue for which the probe must transport across similar biological barriers to reach that tissue. Because nanoparticle accumulation in the liver is predominant after most administration routes [38], we compared intestinal tissue uptake relative to liver uptake as a means to evaluate organ-level specificity of ^64^Cu-NOTA-D500 for GI tract inflammation. The liver is the major xenobiotic metabolizer and was a site of significant ^64^Cu-NOTA-D500 uptake following all administration routes.

After adjusting for distribution to the liver, we observed that oral gavage led to significantly lower probe retention in total intestines and most intestinal tissue regions of DSS mice compared with tissues of Con mice, which was similar to non-significant trends in absolute probe biodistribution. Data and results are shown in Table 2. Unlike oral administration, little difference in liver-normalized intestine tissues was measured between DSS and Con animals after enema administration, with the exception of the cecum.

IP injection was associated with lower liver-normalized probe retention across most tissues in DSS animals compared with Con animals, including within select AT depots (subcutaneous, right gonadal, mesenteric) and individual intestinal tissues (all except mid colon). This outcome reflects the significantly higher uptake of the probe in livers of DSS mice relative to Con mice. Across all three modes of administration, total intestinal uptake of probe relative to the liver was lowest following IP injection. Low intestinal uptake after IP administration likely reflects a lack of direct uptake by the intestines after administration, instead requiring hepatobiliary transport to reach the target tissue. Histologic observations (Figure 2) as well as histopathological reports in other DSS colitis murine models have shown that, although commonly observed in humans, macrophage transepithelial migration and transmural inflammation are only occasionally observed in DSS colitis models [23, 39]. As a result, IP injection would direct the probe toward alternative tissue targets, specifically to the liver, with greater uptake for colitis-affected animals compared with healthy animals.

**Figure 4.**
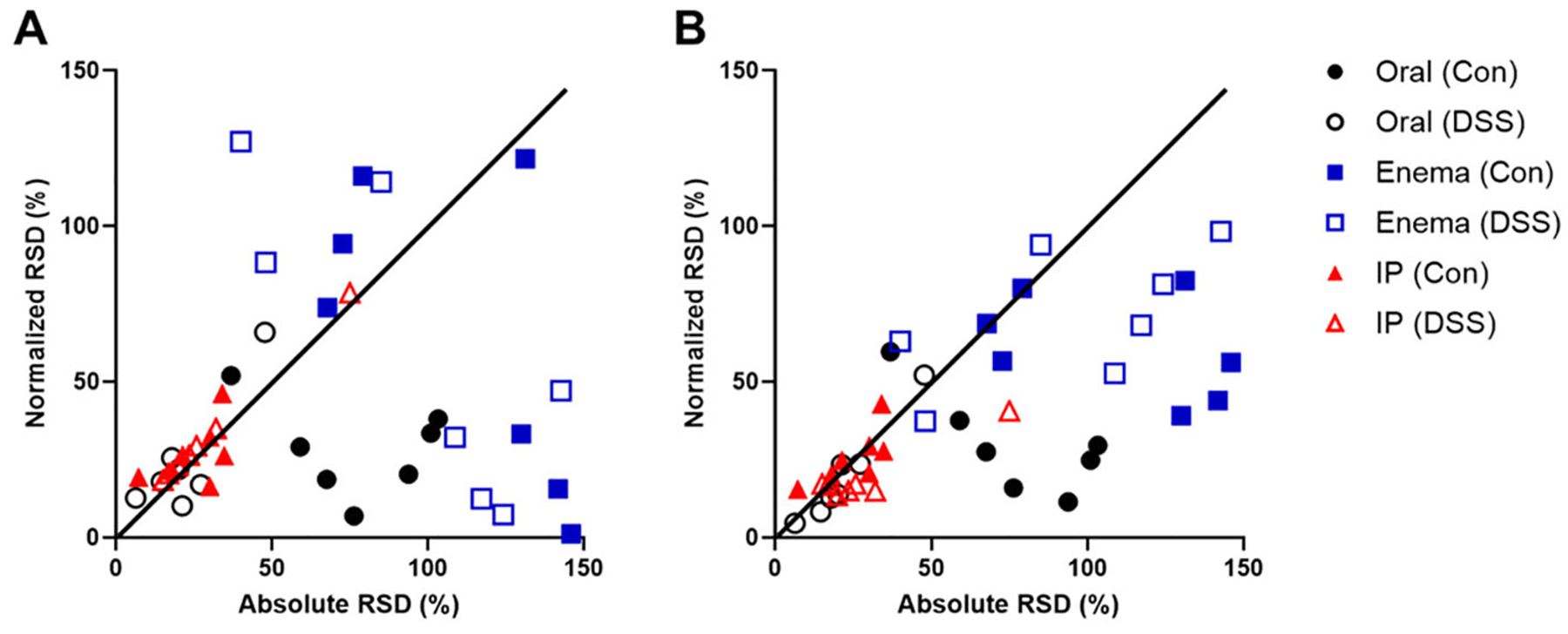
Improvement in inter-subject variability of probe retention in intestinal tissues by normalization. Data points represent the percent relative standard deviation (RSD) with and without normalization to liver or total intestines for 7 intestinal tissues after administration by oral (black circles), enema (blue squares), and IP (red triangles) routes in both healthy (Con) and colitis-affected animals (DSS). Data points below the diagonal line show a reduction in variability due to normalization; those above the vertical line show an increase in variability. (A) Normalization by liver uptake led to improved or worse intestinal tissue RSD for oral and enema administration routes but smaller changes in the RSD for IP administration. (B) Normalization by total intestinal tissue led to reductions in the RSD for oral and enema administration routes, with smaller changes observed for IP administration.

The global impact of liver-based normalization on inter-subject variability is shown in Figure 4A, comparing the relative standard deviation (RSD) of each intestinal tissue with and without normalization. For oral gavage, RSD reduced with liver-based normalization in most tissues, presumably because the quantity of probe entering the intestines distributes proportionally to the hepatic portal system and to further regions of the intestines. In contrast, liver-based normalization after enema administration resulted in mixed outcomes in terms of variability of intestine uptake: RSD for small intestinal tissues and the cecum were strongly reduced; however, RSD for large intestine tissues increased. In addition, a trend of increasing RSD was observed with posterior intestinal position, suggesting that liver normalization is most impactful for the most anterior small intestinal tissues where hepatic uptake occurs. For IP delivery, absolute RSD was smaller overall and uptake normalization by liver had less impact on RSD, although normalization was necessary to observe differences between Con and DSS animals.

### Relative tissue uptake: total intestine normalization

Uptake of ^64^Cu-NOTA-D500 in individual intestinal tissues was evaluated relative to total intestinal retention for each of the administration routes and animal groups (Table 2). As shown in Figure 4B, normalization by total intestines led to a reduction in inter-individual variability for most intestinal tissues for both oral and enema administration routes. With oral administration, DSS animals exhibited significantly greater intestine-normalized probe uptake in tissues of both the small intestine (duodenum, jejunum) and large intestine (proximal and distal colon) compared with Con animals. However, the opposite association was observed for the cecum across all routes of administration. As discussed above, lower probe retention in the cecum of DSS animals may be associated with more efficient clearing from the GI tract due to less intact mucosal lining in some areas of the intestines. In addition, more rapid colonic motility is observed in colitis [40], reducing the time the conjugate spends in the cecum of DSS animals. Although results across modes of administration were not drastically different, both IP and oral administration led to significantly greater relative probe uptake in the duodenum and jejunum compared with enema delivery, while IP injection led to significantly greater uptake by the distal colon compared with oral gavage. As in the case of liver normalization, the RSD was much smaller for IP delivery compared with other administration routes; however, normalization to the total intestines did little to improve group-based differences between Con and DSS animals. Overall, the distribution of probe relative to the total intestines shows that there may be successful targeting of the dextran conjugate within specific intestinal tissues in both colitis-affected and healthy animals when accounting for the total dose that reaches and is retained in the intestines.

### In vivo PET/CT imaging of ^64^Cu-NOTA-D500 biodistribution for three administration routes

**Figure 5.**
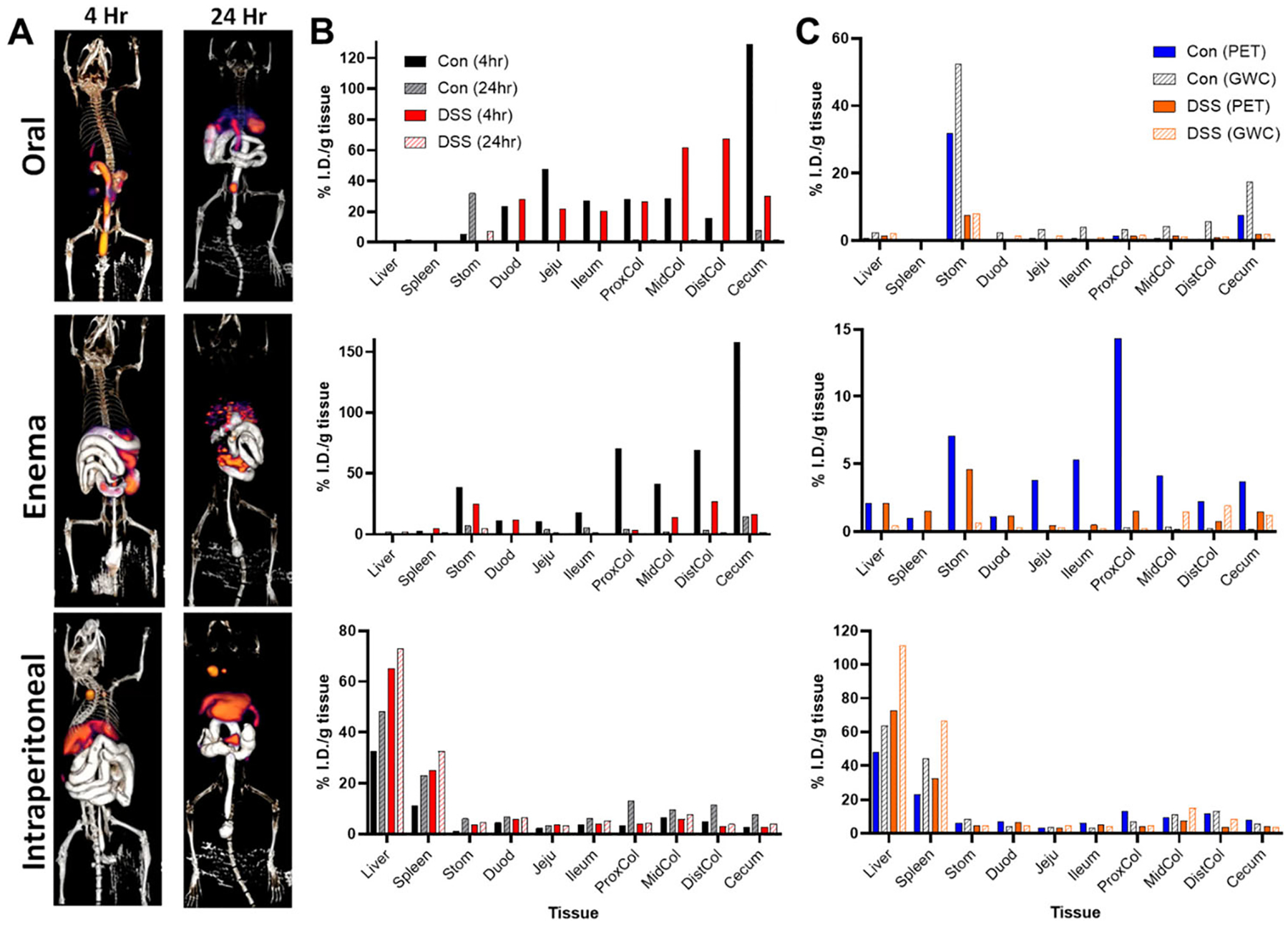
PET quantification of ^64^Cu-NOTA-D500 differs by mode of administration in control (Con) animals and animals with colitis (DSS). (A) Reconstructed PET/CT images 4 and 24 hr following probe administration (oral gavage, enema, or intraperitoneal [IP] injection) with enema-delivered iodine-based contrast to visualize intestines. (B) Total injected dose (I.D.) measured by CT-segmented PET images 4 and 24 hr post-administration (oral, enema, IP) in both Con and DSS animals. (C) Comparison of PET results with gamma well counting (GWC) results 24 hr post-administration. Data are shown as the % injected dose per gram of tissue (I.D./g) for single animal PET/CT imaging and comparative *ex vivo* GWC radioisotopic measurements. Abbreviations: stomach (Stom); duodenum (Duod); jejunum (Jeju); proximal colon (ProxCol); mid colon (MidCol); distal colon (DistCol).

Whole-body PET/CT imaging of ^64^Cu-NOTA-D500 was performed in mice longitudinally at 4 hr and 24 hr following conjugate administration as a comparison to values observed by *ex vivo* GWC. Tissue biodistributions were measured by CT image segmentation of tissues, using iodine-based contrast for intestinal tissues (Figure 5A). Oral gavage led to a distribution pattern that was primarily confined to the GI tract after 4 and 24 hr, consistent with our *ex vivo* GWC studies and similar to previous studies assessing bioavailability of dextran-based oral prodrugs [41]. PET quantification of radioactivity 4 hr after oral administration showed significantly greater probe localization in colon tissues of DSS mice compared with Con mice (Figure 5B), especially relative to total intestines, which was also consistent with *ex vivo* GWC studies. At the 24-hr timepoint, the majority of the probe had cleared, and differences were no longer apparent. Good agreement was observed between PET and *ex vivo* radioisotopic quantification by GWC at the 24 hr time point (Figure 5C), suggesting that oral gavage is an effective route of administration for accurate measurement of intestinal tissue distribution by PET/CT.

PET/CT images displaying probe uptake by tissues following enema administration (Figure 5A) showed predominant localization to the colon, small intestines, and stomach in both Con and DSS mice 4 and 24 hr post-administration (Figure 5B). Significantly higher total probe distribution was measured in the healthy mouse, and as in the oral gavage case, most of the probe had cleared at the 24 hr time point. PET radioisotopic quantification showed poor agreement with *ex vivo* GWC results at the 24 hr time point (Figure 5C) likely due to the significant impact of intestinal contents following enema administration.

IP administration resulted primarily in uptake by the liver and spleen, with less distribution to the colon in both Con and DSS mice at the 4- and 24-hr timepoints (Figure 5A). Consistent with *ex vivo* studies above, PET contrast was significantly higher in livers of DSS mice. Unlike for the case of oral gavage and enema, the vast majority of radioactivity was retained within the body after 24 hr and PET/CT quantification was consistent at 4 and 24 hr post-injection (Figure 5B), providing a large timeframe in which to image. Furthermore, PET radioisotopic quantification closely followed *ex vivo* GWC results across both Con and DSS animals (Figure 5C), suggesting that IP injection may provide the most accurate means for quantifying contrast agent localization.

## Discussion

With the increasing prevalence of IBD worldwide and the deleterious consequences associated with a lack of treatment, it is important to develop diagnostic tools that accurately identify and characterize areas of inflammation associated with IBD. Colonoscopy and endoscopy can visualize limited sections of the intestines, but these methods are invasive and lack repeatability [42]. Although MRI and CT imaging provide a more global assessment of intestinal and extraintestinal inflammation, these methods rely on anatomical contrast alone or non-specific accumulation of contrast agents in areas of active inflammation [43]. Radiolabeled nanomaterials can directly target inflammatory cells and can be visualized by PET to quantitatively assess inflammation severity across independent lesions, improving the specificity of the combined CT imaging modality.

Here, we showed that oral gavage of a radiolabeled dextran conjugate (^64^Cu-NOTA-D500) led to greater uptake by DSS colitis-affected tissues compared with healthy tissues when individual intestinal regions were measured relative to uptake by the total intestines. *In vivo* PET image quantification results followed *ex vivo* GWC results, indicating that oral administration may be preferential for contrast agent visualization of regional gut inflammation. The opposite distribution pattern was observed when intestinal tissues were normalized by the liver, with DSS tissue accumulating less probe than Con tissues. Because the same trend was observed after IP injection, for which DSS animal livers retained nearly two times the quantity of probe compared with Con animals, we attribute this result to altered hepatic function in the case of DSS. Analysis of relative uptake in specific intestinal regions, total intestines, and liver may together be a useful approach for assessing pathological changes across these organ systems *in vivo*.

We also showed that enema administration of ^64^Cu-NOTA-D500 led to significantly greater absolute contrast accumulation within the distal colon of animals affected with DSS colitis compared with healthy control animals. As the primary site of inflammation induced by DSS colitis [23], the distal colon is an appropriate region of interest for evaluating differential probe localization. However, *in vivo* PET/CT measurements were inconsistent with *ex vivo* GWC results, and differences were no longer significant after tissue normalization to total intestines or liver. The inter-subject variability was highest for enema administrations, suggesting that this administration route is inferior to the others, likely due to the intrinsic variability of intestinal contents and possible heterogeneous mechanical effects related to this administration route [37].

When evaluating the biodistribution of ^64^Cu-NOTA-D500 relative to the liver and the intestines, we unexpectedly observed greater probe accumulation within the cecum of Con mice vs. DSS mice across all modes of administration. Con mice should exhibit a more intact mucosa and reduced colonic motility overall throughout the intestines, but there may be less inflammation of this intestinal tissue region in the DSS model compared to other regions [23]. It is also important to consider that reduced intraintestinal pH is commonly observed in IBD patients [44]. Dextran uptake by macrophage lectin receptors such as the mannose receptor is pH-dependent, with binding affinity of both receptors attenuated at pH levels below neutral, physiological values [45–47]. Moreover, pH reductions lead to a closed conformation of the mannose receptor [48, 49], additionally inhibiting dextran uptake by macrophages in a more acidic environment [50].

We also demonstrated that IP delivery of ^64^Cu-NOTA-D500 resulted in higher uptake in most measured tissues in both healthy and DSS colitis mice when compared with oral or enema delivery. While the highest total I.D. of the probe was found in off-target tissues (e.g. visceral AT, liver), absolute retention of the probe by intestinal tissues was much higher 24 hr following IP administration compared with other delivery routes. Notably, *in vivo* measurements remained relatively steady between 4 and 24 hr post administration, and PET measurements were consistent with *ex vivo* GWC results at the 24-hr timepoint. Moreover, inter-subject variability was much less for intestinal tissues after IP injection compared with either oral or enema delivery routes. However, lower liver-normalized contrast within intestinal tissues of DSS animals was observed compared with healthy Con animals, which likely reflects altered liver physiology as opposed to intestinal inflammation. Together with the clinical challenges associated with the use of contrast agents with long retention durations, these results suggest that IP injection is likely to be less effective overall than oral administration.

Histopathological evaluation of colonic tissues indicated that animals with the greatest intestinal damage presented with inflammatory cell infiltration into the submucosa following mucosal collapse. However, there was no evidence of transmural inflammation, as shown in Figure 2. Similar histopathological reports in other DSS colitis murine models have also shown that macrophage transepithelial migration and transmural inflammation are only occasionally observed in DSS colitis models, whereas IBD in humans is commonly associated with transmural inflammation [23, 39]. As such, ^64^Cu-NOTA-D500 contact with the affected luminal tissues of the colon by administration within the peritoneal cavity may be limited by the shallow depth of inflammation with insufficient permeability of the outer muscularis and serosa layers. Previous studies in rats showed a likely molecular weight limit of 45 kDa for permeation into healthy intestinal tissues through the viscera [51]. In models of transmural inflammation with macrophage transepithelial migration, not only may peritoneal macrophages that have taken up the dextran conjugate migrate into the lumen of the colon in response to the inflammatory process, but the outermost tissue layers may be sufficiently damaged to allow dextran direct entry into inflamed tissues. Without this transmural inflammation and damage to the serosal side of the intestines, dextran would be unable to target intestinal inflammation following IP injection. This biological difference is an important point to consider when interpreting results in animal models, as IP administration may lead to greater intestinal uptake in models of transmural inflammation.

Interestingly, like other reports [52, 53], we observed an opposing balance of radiolabel accumulation between the liver and the spleen after IP injection. Within DSS animals, ^64^Cu-NOTA-D500 retention was higher in the liver and lower in the spleen, whereas the opposite effect was seen in Con animals. While the reason for this opposing balance is unknown, differences in conjugate retention in the livers and spleens of Con and DSS mice may be dependent on differences in the biology of the diseased state. With the greatest concentrations of macrophages, the liver and spleen are the primary sites of nanodrug biodistribution [34]; however, higher dextran conjugate uptake by livers in DSS colitis animals compared with liver uptake in Con animals is consistent with greater liver inflammation observed to occur in IBD, in part by immune cell migration directly from the intestines to the liver [54]. This contrast modality could potentially make possible the identification of additional organs affected by inflammation and could be an important diagnostic target for some patients with IBD.

Currently, most available research into nanoparticles for IBD are function-based, examining disease outcome as a marker for nanoparticle delivery [32, 55], and few studies are disease-based, examining the biodistribution of nanoparticles in control vs. diseased tissues [9, 56]. This study sought to examine the route-dependent, disease-based biodistribution of nanoparticles in a murine IBD model, ultimately providing evidence for researchers to apply in considering experimental design, internal normalization parameters, and applying the Reduction principle for animal numbers [57]. We showed that oral administration of a dextran conjugate may lead to differential uptake by colitis-affected tissues when normalizing based on total intestine or liver uptake. Both oral and enema delivery methods led to high inter-individual variability, but internal normalization significantly improved group differences for oral delivery, but not for enema delivery. In contrast, IP delivery led to much lower variability as well as greater absolute uptake of the probe, although this method should be tested in models of transmural inflammation and with lower molecular weight probes.

## Conclusions

Oral and enema delivery of ^64^Cu-NOTA-D500 leads to higher uptake in colitis-affected tissues depending on tissue contrast (liver vs. intestines). Normalized intestinal uptake after oral delivery is the preferred method; however, IP delivery could be preferential for quantification standpoint if transmural inflammation is present, as is often seen in humans. Oral and IP delivery may additionally diagnose liver changes, affecting about 50% of all individuals afflicted with IBD. Results here suggest that oral delivery with appropriate normalization may be the best route of administration for quantitative imaging in biologically variable IBD models, which should guide research design towards applying the Reduction principle of the 3 R’s of animal research (Replace, Reduce, Refine).

## Supplementary Material

Applegate_Supplemental

## Figures and Tables

**Table 1.** Tissue biodistribution of radiolabeled dextran conjugate by mode of administration.^a^

	Oral			Enema			Intraperitoneal			Across modes *p*^c^
Tissue	Control	DSS	*p* ^b^	Control	DSS	*p* ^b^	Control	DSS	*p* ^b^	
AT-SubQ	0.09 ± 0.04	0.18 ± 0.05	0.337	0.04 ± 0.01	0.08 ± 0.06	0.512	1.79 ± 0.69	0.79 ± 0.15	0.231	**<0.0001** * ^0^
AT-L Gonad	0.03 ± 0.01	0.09 ± 0.03	0.218	0.06 ± 0.03	0.03 ± 0.01	0.358	75.9 ± 11.5	64.7 ± 16.5	0.608	**0.0002** * ^0^
AT-R Gonad	0.03 ± 0.01	0.09 ± 0.03	0.212	0.02 ± 0.01	0.16 ± 0.13	0.341	56.1 ± 4.06	53.3 ± 16.5	0.878	**0.0005** * ^0^
AT-L PR	0.08 ± 0.05	0.27 ± 0.06	0.125	0.13 ± 0.02	0.92 ± 0.77	0.362	25.6 ± 2.40	132.6 ± 84.6	0.275	**0.0005** * ^0^
AT-R PR	0.11 ± 0.03	0.38 ± 0.15	0.254	0.13 ± 0.02	0.84 ± 0.69	0.362	31.1 ± 4.73	82.9 ± 24.9	0.111	**0.0006** * ^0^
AT-Mes	0.10 ± 0.07	0.23 ± 0.05	0.260	0.05 ± 0.01	0.03 ± 0.02	0.490	34.1 ± 2.07	34.5 ± 7.63	0.962	**<0.0001** * ^0^
AT-Total	0.45 ± 0.21	1.23 ± 0.33	0.190	0.44 ± 0.08	2.07 ± 1.58	0.362	224.5 ± 14.0	368.7 ± 102.3	0.235	**0.0004** * ^0^
Liver	1.57 ± 0.90	2.52 ± 0.26	0.294	0.33 ± 0.28	0.82 ± 0.59	0.489	65.0 ± 5.76	116.0 ± 2.29	**0.001**	**<0.0001** * ^0^
Spleen	0.14 ± 0.01	0.68 ± 0.19	0.122	0.06 ± 0.04	0.15 ± 0.05	0.230	114.6 ± 35.9	50.3 ± 11.5	0.163	**<0.0001** * ^0^
Stomach	27.2 ± 25.1	3.94 ± 2.04	0.306	1.00 ± 0.84	2.98 ± 2.64	0.515	6.54 ± 0.96	5.50 ± 0.61	0.413	**0.085** ^0^
Duod	1.57 ± 0.85	1.67 ± 0.14	0.888	0.26 ± 0.22	0.43 ± 0.27	0.658	5.07 ± 0.52	3.78 ± 0.37	0.115	**<0.0001** *^0†^
Jeju	1.94 ± 1.38	1.75 ± 0.20	0.874	0.23 ± 0.18	0.51 ± 0.35	0.506	4.16 ± 0.17	3.44 ± 0.64	0.336	**<0.0001** *^0†^
Ileum	2.38 ± 1.74	1.25 ± 0.20	0.455	0.32 ± 0.13	0.45 ± 0.32	0.730	5.09 ± 1.00	3.48 ± 0.30	0.199	**0.0001** ^0 †^
Prox Colon	1.78 ± 0.85	1.66 ± 0.17	0.866	0.40 ± 0.18	1.00 ± 0.49	0.320	7.10 ± 1.23	3.76 ± 0.56	**0.069**	**<0.0001** * ^0^
Mid Colon	1.38 ± 0.58	1.42 ± 0.17	0.936	0.50 ± 0.38	1.42 ± 0.39	0.166	8.53 ± 1.48	8.10 ± 3.51	0.915	**0.0002** * ^0^
Dist Colon	0.93 ± 0.24	1.27 ± 0.05	0.169	0.24 ± 0.10	1.60 ± 0.37	**0.024**	11.2 ± 2.24	6.93 ± 0.79	0.149	**0.0004** * ^0^
Cecum	10.5 ± 6.96	1.29 ± 0.36	0.175	2.44 ± 1.83	0.95 ± 0.78	0.496	7.57 ± 0.93	3.14 ± 0.43	**0.013**	**0.068** ^0^
Total Intest	20.5 ± 12.6	10.3 ± 0.36	0.359	4.39 ± 2.99	6.36 ± 2.67	0.649	48.7 ± 2.86	32.6 ± 6.36	**0.083**	**0.0007** * ^0^

Abbreviations: AT: adipose tissue; SubQ: subcutaneous; L/R Gonad: left/right gonadal; L/R PR: left/right perirenal; Mes: mesenteric; Duod: duodenum; Jeju: jejunum; Intest: intestine.

a Data are expressed as mean ± SEM % injected dose.

b Significant values (*p* < 0.05 and *p* < 0.10 for trend) are indicated in bold; Wilcoxon Rank Sum comparing Con vs. DSS tissue biodistribution; *n* = 3/group.

c Significant values are indicated in bold; Kruskal-Wallis comparing total (Con + DSS) tissue biodistribution between modes of administration; *n* = 6/group.

* IP significantly different from oral.

0 IP significantly different from enema.

† Oral significantly different from enema.

**Table 2.** Tissue biodistribution by mode of administration relative to liver and total intestinal uptake of radiolabeled dextran conjugate.^a^

		Oral			Enema			Intraperitoneal			Across modes *p*^c^
Tissue	Relative tissue	Control	DSS	*p* ^b^	Control	DSS	*p* ^b^	Control	DSS	*p* ^b^	
AT-SubQ	Liver	0.01 ± 0.01	0.07 ± 0.02	0.841	0.61 ± 0.29	0.17 ± 0.08	0.216	0.03 ± 0.01	0.01 ± 0.00	**0.097**	**0.001** * ^0^
AT-L Gonad		0.03 ± 0.01	0.03 ± 0.01	0.572	0.90 ± 0.47	0.24 ± 0.22	0.270	1.22 ± 0.29	0.56 ± 0.16	0.116	**0.023** *
AT-R Gonad		0.02 ± 0.01	0.03 ± 0.01	0.461	0.46 ± 0.23	2.13 ± 2.08	0.470	0.87 ± 0.09	0.47 ± 0.15	**0.082**	**0.019** *
AT-L PR		0.05 ± 0.00	0.10 ± 0.02	0.115	1.63 ± 0.74	12.5 ± 12.3	0.427	0.40 ± 0.06	1.13 ± 0.71	0.366	**0.006** * ^†^
AT-R PR		0.09 ± 0.03	0.14 ± 0.05	0.456	2.06 ± 1.03	11.3 ± 11.1	0.454	0.49 ± 0.08	0.71 ± 0.21	0.367	**0.022** *
AT-Mes		0.06 ± 0.01	0.09 ± 0.01	0.236	0.56 ± 0.29	0.37 ± 0.35	0.695	0.54 ± 0.08	0.30 ± 0.07	**0.089**	**0.097** *
AT-Total		0.31 ± 0.05	0.47 ± 0.09	0.297	6.22 ± 2.92	26.7 ± 26.1	0.479	3.54 ± 0.50	3.18 ± 0.86	0.734	**0.022** *
Spleen		0.13 ± 0.07	0.26 ± 0.06	0.261	0.20 ± 0.18	0.58 ± 0.40	0.454	1.81 ± 0.64	0.44 ± 0.11	0.101	**0.043** *
Duod		1.03 ± 0.05	0.68 ± 0.07	**0.036**	0.80 ± 0.01	0.68 ± 0.13	0.395	0.08 ± 0.01	0.03 ± 0.00	**0.012**	**0.0005** * ^0^
Jeju		1.09 ± 0.26	0.71 ± 0.09	0.188	0.77 ± 0.07	0.69 ± 0.05	0.395	0.07 ± 0.01	0.03 ± 0.01	**0.020**	**0.0003** * ^0^
Ileum		1.31 ± 0.35	0.49 ± 0.05	**0.057**	2.77 ± 1.18	0.53 ± 0.02	0.131	0.08 ± 0.02	0.03 ± 0.00	**0.081**	**0.0004** * ^0^
Prox Col		1.23 ± 0.16	0.68 ± 0.10	**0.052**	4.14 ± 2.78	1.49 ± 0.98	0.439	0.11 ± 0.01	0.03 ± 0.01	**0.003**	**0.0004** * ^0^
Mid Col		1.00 ± 0.21	0.57 ± 0.03	**0.072**	2.85 ± 2.01	4.98 ± 2.54	0.547	0.13 ± 0.02	0.07 ± 0.03	0.202	**<0.0001** * ^0^
Dist Colon		0.75 ± 0.28	0.51 ± 0.04	0.343	2.34 ± 1.27	7.58 ± 5.57	0.411	0.17 ± 0.03	0.06 ± 0.01	**0.015**	**<0.0001** * ^0 †^
Cecum		6.19 ± 0.89	0.55 ± 0.21	**0.004**	9.71 ± 1.86	1.13 ± 0.31	**0.011**	0.12 ± 0.02	0.03 ± 0.00	**0.008**	**0.0002** * ^0^
Total Intest		12.6 ± 0.81	4.18 ± 0.40	**0.002**	23.4 ± 6.72	17.1 ± 7.92	0.577	0.75 ± 0.02	0.28 ± 0.06	**0.002**	**<0.0001** * ^0^
Duod	Total Intest	0.08 ± 0.01	0.16 ± 0.01	**0.007**	0.04 ± 0.01	0.05 ± 0.02	0.601	0.10 ± 0.01	0.12 ± 0.01	0.411	**0.002** ^0 †^
Jeju		0.09 ± 0.01	0.17 ± 0.01	**0.027**	0.04 ± 0.01	0.06 ± 0.02	0.435	0.09 ± 0.01	0.11 ± 0.01	0.174	**0.013** ^0 †^
Ileum		0.10 ± 0.02	0.12 ± 0.02	0.557	0.11 ± 0.04	0.05 ± 0.02	0.290	0.11 ± 0.03	0.11 ± 0.01	0.892	0.323
Prox Col		0.10 ± 0.02	0.16 ± 0.01	**0.061**	0.14 ± 0.07	0.13 ± 0.07	0.928	0.14 ± 0.02	0.12 ± 0.01	0.282	0.873
Mid Col		0.08 ± 0.02	0.14 ± 0.02	0.141	0.11 ± 0.05	0.26 ± 0.06	0.119	0.18 ± 0.03	0.23 ± 0.05	0.443	0.150
Dist Colon		0.06 ± 0.03	0.12 ± 0.00	**0.052**	0.08 ± 0.03	0.33 ± 0.12	0.116	0.23 ± 0.04	0.22 ± 0.02	0.864	**0.019** *
Cecum		0.49 ± 0.04	0.13 ± 0.04	**0.008**	0.47 ± 0.11	0.10 ± 0.06	**0.040**	0.16 ± 0.02	0.10 ± 0.01	**0.072**	0.561

Abbreviations: AT: adipose tissue; SubQ: subcutaneous; L/R Gonad: left/right gonadal; L/R PR: left/right perirenal; Mes: mesenteric; Duod: duodenum; Jeju: jejunum; Prox Col: proximal colon; Mid Col: mid colon; Dist Col: distal colon; Intest: intestine.

a Data are expressed as mean ± SEM % injected dose.

b Significant values (*p* < 0.05 and *p* < 0.10 for trend) are indicated in bold; Wilcoxon Rank Sum comparing Con vs. DSS tissue biodistribution; *n* = 3/group.

c Significant values are indicated in bold; Kruskal-Wallis comparing total (Con + DSS) tissue biodistribution between modes of administration; *n* = 6/group.

* IP significantly different from oral.

0 IP significantly different from enema.

† Oral significantly different from enema.

## Data Availability

The data that support the findings of this study are available from the corresponding author, KSS and AMS, upon reasonable request.
