# Supplementary material for: Impact of administration route on nanocarrier biodistribution in a murine colitis model
Source: J Exp Nanosci. Author manuscript; Available in PMC 2023 Oct 19. (PMC10038121; doi:10.1080/17458080.2022.2134563)
Supplement: Applegate_Supplemental [file NIHMS1860150-supplement-Applegate_Supplemental.docx]

**Impact of Administration Route on Nanocarrier Biodistribution in a Murine Colitis Model**

*Catherine C. Applegate^1,2^, Hongping Deng^3^, Brittany L. Kleszynski^4^, Tzu-Wen L. Cross^5^, Christian J. Konopka^5,6^, L. Wawrzyniec Dobrucki^5,6,7^, Erik R. Nelson^1,7,8,9,10^, Matthew A. Wallig^1,3^, Andrew M. Smith^3,7,11,12,13^*, Kelly S. Swanson^1,2^**

**Supplementary Information**

^1^Division of Nutritional Sciences, University of Illinois at Urbana−Champaign, Urbana, IL 61801, USA.

^2^Department of Animal Sciences, University of Illinois at Urbana−Champaign, Urbana, IL 61801, USA.

^3^Department of Bioengineering, University of Illinois at Urbana−Champaign, Urbana, IL 61801, USA.

^4^Department of Pathobiology, College of Veterinary Medicine, University of Illinois at Urbana−Champaign, Urbana, IL 61801, USA.

^5^Department of Nutrition Science, Purdue University, West Lafayette, IN 47907, USA.

^6^Beckman Institute for Advanced Science and Technology, Urbana, IL 61801, USA.

^7^Cancer Center at Illinois, University of Illinois at Urbana−Champaign, Urbana, IL 61801, USA.

^8^Department of Molecular and Integrative Physiology, University of Illinois at Urbana−Champaign, Urbana, IL 61801, USA.

^9^University of Illinois Cancer Center, University of Illinois at Chicago, Chicago, IL 60612, USA.

^10^Carl R. Woese Institute for Genomic Biology, Anticancer Discovery from Pets to People Theme, University of Illinois Urbana-Champaign, Urbana, IL 61801, USA.

^11^Carle Illinois College of Medicine, Urbana, Illinois 61801, USA.

^12^Micro and Nanotechnology Laboratory, University of Illinois at Urbana−Champaign, Urbana, IL 61801, USA.

^13^Department of Materials Science and Engineering, University of Illinois at Urbana−Champaign, Urbana, IL 61801, USA.


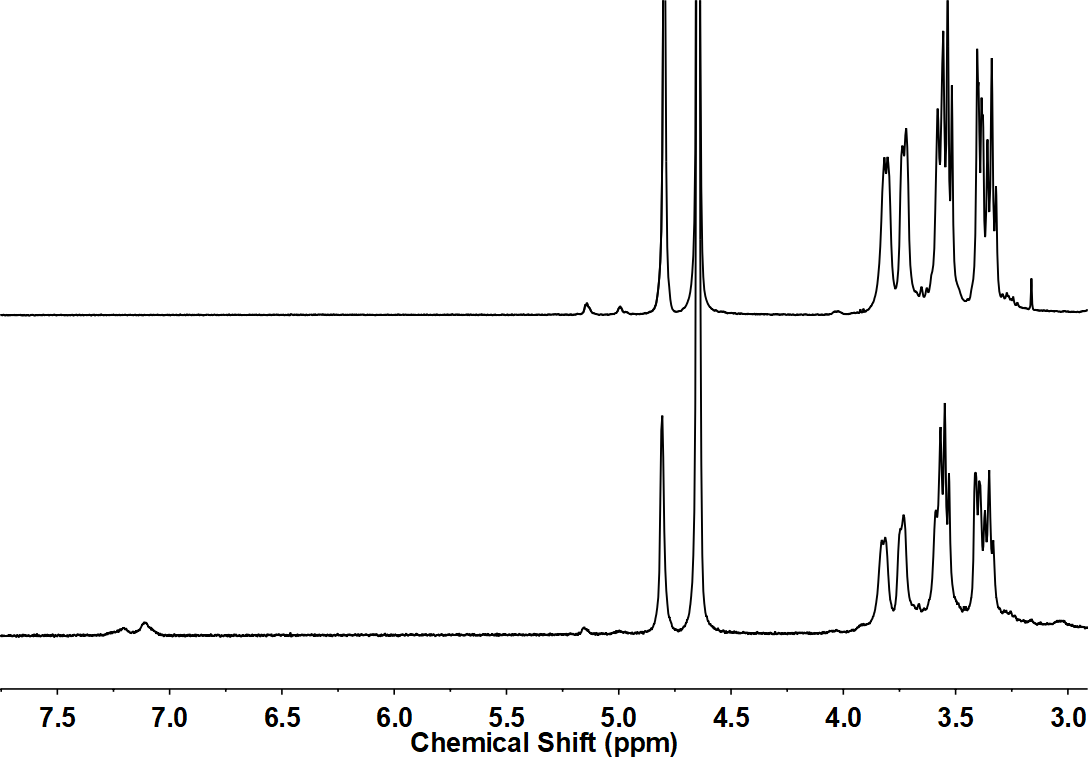


**Figure S1. Proton nuclear magnetic resonance (^1^H NMR) spectroscopy depicting conjugation of NOTA.** The appearance of peaks at 7.0-7.3 ppm (lower image) were consistent with NOTA, confirming conjugation to the probe.
